# Primary ciliary dyskinesia caused by a large homozygous deletion including exons 1–4 of *DRC1* in Japanese patients with recurrent sinopulmonary infection

**DOI:** 10.1002/mgg3.1033

**Published:** 2019-11-08

**Authors:** Naoto Keicho, Minako Hijikata, Kozo Morimoto, Sakae Homma, Yoshio Taguchi, Arata Azuma, Shoji Kudoh

**Affiliations:** ^1^ The Research Institute of Tuberculosis Japan Anti‐Tuberculosis Association Tokyo Japan; ^2^ Department of Pathophysiology and Host Defense the Research Institute of Tuberculosis Japan Anti‐Tuberculosis Association Tokyo Japan; ^3^ Respiratory Disease Center Fukujuji Hospital Japan Anti‐Tuberculosis Association Tokyo Japan; ^4^ Department of Advanced and Integrated Interstitial Lung Diseases Research School of Medicine Toho University Tokyo Japan; ^5^ Department of Respiratory Medicine Tenri Hospital Nara Japan; ^6^ Division of Pulmonary Medicine and Oncology, Graduate School of Medicine Nippon Medical School Tokyo Japan; ^7^ Japan Anti‐Tuberculosis Association Tokyo Japan

**Keywords:** diffuse panbronchiolitis, founder mutation, loss‐of‐function variant, nexin‐dynein regulatory complex, primary ciliary dyskinesia, sinopulmonary disease

## Abstract

**Background:**

Diffuse panbronchiolitis (DPB) is a sinopulmonary disease mainly affecting Asian populations. Primary ciliary dyskinesia (PCD) is a genetically heterogeneous disorder impairing ciliary structure and function. These two disorders are not easily distinguished by clinical signs and symptoms.

**Methods:**

In 105 Japanese patients with recurrent sinopulmonary infection, initially diagnosed with DPB, and 37 patients with recurrent airway infection diagnosed in adulthood, the deletion allele of *DRC1* or *CCDC164*, recently recognized as a pathogenic PCD gene variant, was searched using a multiplexed PCR‐based method, and the deletion breakpoints and other variants around the gene were determined by Sanger sequencing and targeted resequencing.

**Results:**

A large homozygous deletion in *DRC1* was identified in three of the 142 patients. Furthermore, heterozygous carriers of the deletion with the same breakpoint were found with the allele frequency of 0.002 in the healthy Japanese population, indicating that this loss‐of‐function variant may be acting as a common mutation causing PCD in Japanese.

**Conclusion:**

PCD caused by the *DRC1* defect is not readily identified by either high‐speed video‐microscopy or ciliary ultrastructure analysis, posing significant difficulties in reaching a correct diagnosis without the aid of genetic tests. Careful investigation of the causes of sinopulmonary diseases is warranted in Asian populations.

## Graphical abstract

A large homozygous deletion in *DRC1* was identified in three patients with recurrent sinopulmonary infection. Furthermore, heterozygous carriers of the *DRC1* deletion with the same break point were found with the allele frequency of 0.002 in the healthy Japanese population, indicating that this loss‐of‐function variant may be acting as a common mutation causing PCD in Japanese.

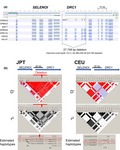

## 1 INTRODUCTION

Diffuse panbronchiolitis (DPB) is a clinical entity characterized by sinopulmonary infection with a large amount of sputum production, and small inflammatory nodular lesions around the respiratory bronchioles in the lung (Homma et al., 1983). In a survey, the prevalence of DPB was estimated to be 13.8 and 6.6 in 100,000 in the 1980s and 1990s, respectively (Kono et al., 2012). DPB, a multifactorial disease, is associated with human leukocyte antigen (HLA) class‐I alleles. It affects mainly Asian populations, including Japan, Korea, and China (Ding et al., 2007; Keicho & Hijikata, 2011; Park et al., 1999), and is distinct from cystic fibrosis (CF) in encountered European descents.

Primary ciliary dyskinesia (PCD) is a genetically heterogeneous disorder of the ciliary structure and function (Afzelius, 1976; Lucas et al., 2017; Shapiro et al., 2016), and follows both autosomal recessive and X‐linked inheritance (Olcese et al., 2017). It is estimated to affect one in 15,000–30,000 individuals in the world (Bush et al., 2007). Approximately 40 genes that cause PCD have been identified so far. Its classical form with a triad of situs inversus, chronic sinusitis, and bronchiectasis, is known as Kartagener syndrome. Sinopulmonary diseases including PCD, DPB and CF, are characterized by leading symptoms such as productive cough, chronic rhinosinusitis, and recurrent infections of the upper and lower respiratory tract; these diseases are not easily distinguished clinically from each other in case of situs solitus, the normal position of organs (Amitani, Tomioka, Kurasawa, Ishida, & Kuze, 1990; Knowles, Zariwala, & Leigh, 2016).

Recently, we experienced an adult patient, who was followed based on initial diagnosis, as DPB. However, eventually, a homozygous loss‐of‐function mutation within *DRC1* (*CCDC164*, MIM 615288), one of the PCD causative genes, was identified in the patient, who was re‐diagnosed with PCD (Morimoto et al., 2019). The first case of PCD caused by a *DRC1* mutation was reported only six years ago (Wirschell et al., 2013). These findings prompted us to search for the same variants in DNA samples obtained from patients previously diagnosed as having DPB in Japan.

## 2 MATERIALS AND METHODS

The ethical committee approved the study protocol (RIT/IRB 29–28) at the Research Institute of Tuberculosis of the Japanese Anti‐Tuberculosis Association, and written informed consent was obtained from each individual for the purpose of disease gene research. Demographic and clinical information of the patients has been extracted from the metadata, retrospectively (Hijikata et al., 2011).

### 2.1 DNA and clinical information about patients

DNA samples were available from 105 of 108 unrelated Japanese patients previously reported by Hijikata et al. (Hijikata et al., 2011). All fulfilled the following diagnostic criteria for DPB proposed in 1995 by a working group of the Ministry of Health and Welfare of Japan: persistent cough, sputum, and exertional dyspnea; history of, or current chronic sinusitis; bilateral diffuse small nodular shadows on a plain chest X‐ray film or centrilobular micronodules on chest computed tomography (CT) images; coarse crackles; FEV_1.0_/FVC less than 70% and PaO_2_ less than 80 Torr; and titer of cold hemagglutinin equal to or higher than 64 ×. Definite cases should fulfill the first three criteria and at least two other remaining criteria, excluding other chronic respiratory diseases (Keicho & Kudoh, 2002). Our screening was extended to 37 patients with recurrent infections of the upper and lower airways, recruited in the same period (mainly from 1997 to 1999), but not meeting the DPB criteria. The DNA panel of the healthy anonymous Japanese population, consisting of 499 samples was obtained from the Health Science Research Resources Bank (Japan Health Sciences Foundation).

### 2.2 PCR to detect a large deletion spanning exons 1–4 in *DRC1*

Genomic DNA was tested for the presence or absence of the *DRC1* deletion allele using the multiplexed PCR‐based method as described recently (Morimoto et al., 2019). Briefly, the deletion allele was detected in a 515‐bp fragment using primers located outside the deletion, and the wild type allele was amplified in 600‐bp, 376‐bp, and 849‐bp fragments with primer pairs from flanking regions of exons 2, 3, and 4, respectively. PCR products were electrophoresed in 2% agarose gel and stained with GelRed (Biotium).

### 2.3 Sanger sequencing to confirm nucleotide positions of deletion junctions

The genomic region encompassing the deletion in *DRC1* was amplified with primers 5′‐CAGTGGACTAAGGTAGGTGCTTGG‐3′ and 5′‐ACCAAATGGCTTTGACAAGGGTCA‐3′, with Tks Gflex DNA polymerase (TaKaRa) as recommended by the manufacturer, and the PCR conditions were 94ºC for 1 min followed by 35 cycles of 98ºC for 10 s, 60ºC for 15 s, and 68ºC for 1 min 30 s. The PCR product of 1,215 bp was purified with QIAquick PCR purification kit (QIAGEN) and sequenced bidirectionally with primers 5′‐GGCAGTCAGTCCTACATATCAAGG‐3′ or 5′‐TAAGAAGGCAATATACGGAATAGG‐3′, using BigDye terminator v3.1 cycle sequencing kit and SeqStudio Genetic Analyzer (Thermo Fisher Scientific).

### 2.4 Targeted resequencing for all exons of *DRC1* and *SELENOI* genes

Seventeen exons of *DRC1* and adjacent 10 exons of *SELENOI* were amplified by PCR using 15 primer sets (Table S1). In each analyzed patient or control, the PCR products were mixed, purified with QIAquick PCR purification kit and subjected to library preparation using QIAseq FX DNA Library Kit (QIAGEN). The appropriate library size was selected with AMPure XP beads (Beckman Coulter), and 2 × 150 bp paired‐end sequencing run was performed using MiSeq Reagent Kit Nano v2 (300 Cycle) and MiSeq Next‐Generation Sequencing System (Illumina). The sequence reads were trimmed, aligned to the human genome (hg38), and variants were called and listed using CLC Genomics Workbench version 11.0 (QIAGEN). Genotype data of Japanese in Tokyo (JPT) and Northern and Western European Ancestry (CEU) populations were downloaded from the 1,000 genomes project (http://www.1000genomes.org). Haplotypes consisting of the variants in exon regions of *DRC1* and *SELENOI* were estimated using Haploview 4.2 (Barrett, Fry, Maller, & Daly, 2005).

## 3 RESULTS

### 3.1 Characteristics of the patients meeting the clinical diagnostic criteria for DPB

Demographic data are shown in Table 1. All of the 105 patients, reported previously (Hijikata et al., 2011), fulfilled the clinical diagnostic criteria for DPB described in the Materials and Methods. Raw data of clinical and demographic information about all the patients are shown in Table S2.

**Table 1:** Characteristics of the patients meeting the clinical diagnostic criteria for DPB

Patients	(*N* = 105)
Male/Female (*n*)	58/46^a^
Age at diagnosis (year)	52 (41–62)^b^
Duration from diagnosis to recruitment (year)	6 (2–11)^b^
Persistent cough, sputum, and exertional dyspnea (*n*)	105
History of or current chronic sinusitis (*n*)	105
Bilateral diffuse small nodular shadows (*n*)	105 (only on CT images = 10)
FEV_1.0_/FVC (%)	60.0 (46.7–66.7)^b^
PaO_2_ (Torr)	68.0 (60.2–74.0)^b^
Cold hemagglutinin (titer)	512 × (128 × −1,024 ×)^b^
HLA‐B*5401 (*n*)	39 (monoallelic = 35, biallelic = 4)
HLA‐B*5504 (*n*)	4 (monoallelic = 4)

a one missing.

b Median and 25 to 75 percentiles in parenthesis.

### 3.2 Detection of loss‐of‐function mutations of the *DRC1* gene in patients

**Figure 1:**
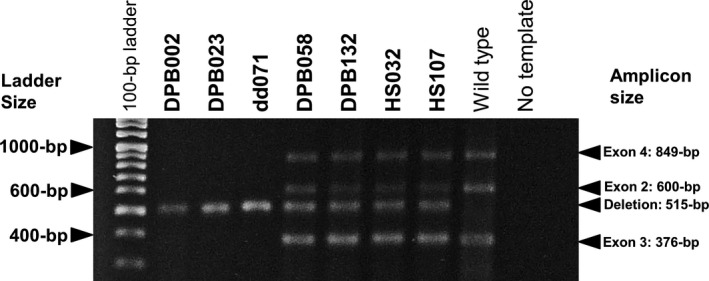
Detection of PCR products from the multiplexed PCR‐based method by agarose gel electrophoresis. Only deletion alleles were amplified from genomic DNA samples of two DPB patients (DPB002 and DPB023) and of a patient with recurrent airway infections (dd071). Both wild type and deletion alleles were amplified from those of two other DPB patients (DPB058 and DPB132) and two healthy individuals (HS032 and HS107). One reference sample showing homozygous insertion (wild type) and PCR products with no genomic DNA (No template) were also shown as positive and negative controls, respectively

Using a multiplexed PCR‐based method, the presence or absence of a large homozygous deletion spanning exons 1–4 of *DRC1* was detected. Of the 105 patients with DPB, two were homozygous for the deletion allele (Figure 1: DPB002 and DPB023), and two others had heterozygous deletion alleles (Figure 1: DPB058 and DPB132). For these four patients, no other pathogenic or likely pathogenic variants were found in any exons and exon‐intron boundaries of *DRC1* by targeted resequencing and Sanger sequencing (Table S3).

### 3.3 Clinical signs and symptoms of the patients carrying the large deletion in the *DRC1* gene

As shown in Table 2, all of the patients had typical signs and symptoms as sinopulmonary disease, and the former two patients' age at diagnosis was 43 and 29 years old. The two patients did not have the HLA‐B*5401 or HLA‐B*5504 alleles, known as DPB susceptibility alleles (Keicho et al., 1998). These *HLA‐B* alleles are known to be disease markers for DPB, and a major disease susceptibility gene is suspected to be located near the *HLA‐B* locus on human chromosome 6 (Hijikata et al., 2011; Keicho et al., 2000). In addition, a cold hemagglutinin test that often shows high titers in DPB (Sugiyama, Kudoh, Maeda, Suzaki, & Takaku, 1990) was low or borderline in these patients. Consanguinity was not described. Situs inversus was not recorded. Although a long‐term macrolide treatment was effective in most of the 105 patients, according to the description by doctors‐in‐charge, the treatment effect such as an improvement in signs and symptoms, including sputum production, was not substantial in the second homozygous patient (DPB023), showing diffuse centrilobular micronodules with focal bronchiectasis.

**Table 2:** Clinical signs and symptoms of the patients homozygous (DPB002 and DPB023) and heterozygous (DPB058 and DPB132) for the large deletion in the *DRC1* gene

	DPB002	DPB023	DPB058	DPB132
Male/Female	Female	Female	Female	Male
Age at diagnosis (year)	43	29	43	62
Duration from diagnosis to recruitment (year)	0	3	26	1
Persistent cough, sputum, and exertional dyspnea	Yes	Yes	Yes	Yes
History of or current chronic sinusitis	Yes	Yes	Yes	Yes
Bilateral diffuse small nodular shadows	Yes	Yes	Yes	Yes
FEV_1.0_/FVC (%) (normal range ≥70%)	43.0	69.1	53.0	70.9
PaO_2_ (Torr) (normal range ≥80 Torr)	71	NA	64	82
Cold hemagglutinin (titer) (normal range <64 ×)	4 ×	64 ×	NA	128 ×
HLA‐B alleles	HLA‐B*4801/HLA‐B*5101	HLA‐B*4801/HLA‐B*5201	HLA‐B*0702/HLA‐B*5401	HLA‐B*0701/HLA‐B*5401
Large *DRC1* deletion	homozygous	homozygous	heterozygous	heterozygous

Abbreviations: NA, not available.

Of the 37 patients with recurrent infections of the upper and lower airways recruited in the same period but not meeting the DPB criteria, another 41‐year‐old male patient also had the homozygous deletion in *DRC1* (Figure 1: dd071); however, detailed information about this patient was not obtained.

### 3.4 Allele frequency distribution of the large *DRC1* deletion in healthy Japanese, and confirmation of the deletion junctions

Carriers of the identical genetic deletion were found in two of 499 healthy Japanese (Figure 1: HS032 and HS107). An estimated allele frequency in the Japanese population of the large deletion in *DRC1* was, thus, 0.002.

**Figure 2:**
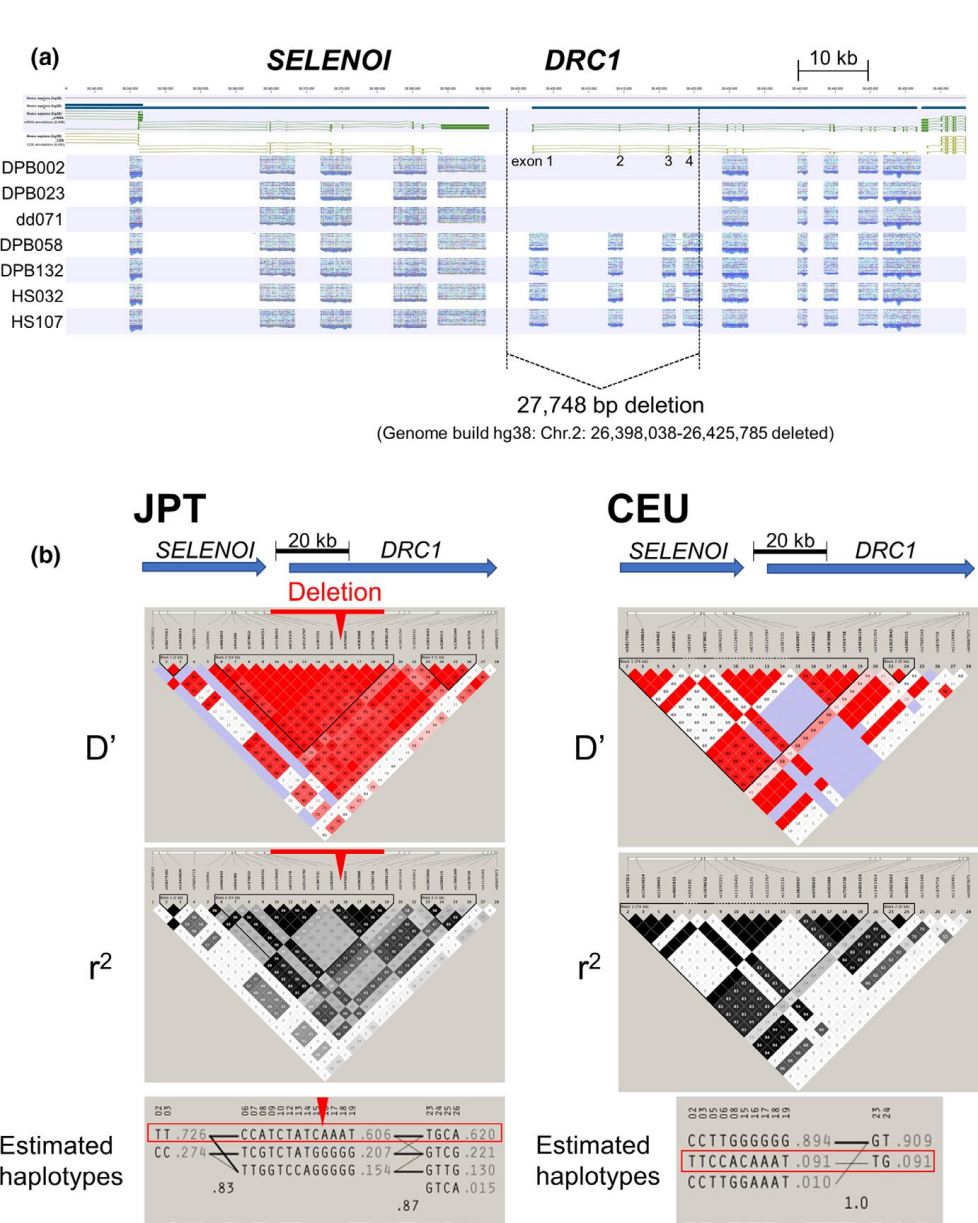
Recurring large deletions in *DRC1* (a) A targeted resequencing profile around *SELENOI* and *DRC1*. The first three lanes (DPB002, DPB023, and dd071) indicate large homozygous deletions spanning exons 1–4 of *DRC1*. The rest (DPB058, DPB132, HS032, and HS107) had heterozygous deletions, and PCR products were thus mapped to all exons because of the presence of the insertion allele. Deletion breakpoints are illustrated as dotted lines within the *SELENOI‐DRC1* intergenic region and within intron 4 of *DRC1.* The deletion size between the dotted lines was 27,848 bp, according to hg38. (b) The D', r^2^ values and haplotype estimation using JPT (left) and CEU (right) data of the positions listed in Table S4. The deletion in *DRC1* was carried by the major haplotype (in the red box) of JPT. The same haplotype was also found in the CEU population but with a reduced frequency than in the JPT

To identify the 27,748 bp deletion, the PCR products encompassing the deletion were directly sequenced by the Sanger sequencing method, with breakpoints in *SELENOI*‐*DRC1* intergenic region and intron 4 of *DRC1*, identical to the deletion found in our initially reported cases (Morimoto et al., 2019), and shared among all deletion alleles, including those of two healthy carriers (Figure S1). An overall profile of targeted resequencing of the above seven cases is illustrated in Figure 2a, and observed variations are summarized in Table S4. Three patients with the homozygous deletions (six alleles in total) carried the same homozygous genotypes for all the variations listed in Table S4; therefore, only one haplotype could be estimated from them. The haplotype estimation using JPT data of the listed positions in Table S4 revealed that the deletion was carried by a major haplotype of the JPT population, as shown in Figure 2b. The same haplotype was also expected in the CEU population. However, the estimated frequency (9.1%) in this population was much lower than that observed in the JPT (60.1%).

## 4 DISCUSSION

We identified a large homozygous deletion in *DRC1* in two (1.9%) of 105 Japanese adult patients clinically diagnosed as having DPB in the past and a heterozygous deletion in two other patients, in which other loss‐of‐function mutations have not yet been found within our search. During the same period, another Japanese patient with recurrent airway infection was also found to have the homozygous deletion in *DRC1*. Surprisingly, carriers of the large deletion with the same breakpoint were found with the allele frequency of 0.002, which was observed in a major haplotype in the Japanese population. However, the same deletions were not found in more than 20,000 non‐Asians (Morimoto et al., 2019), indicating that this loss‐of‐function variant may be a major cause of PCD in Japan, presumably acting as a founder mutation. Clinical features of these patients are hardly distinguishable from those of classical DPB, posing significant difficulties in reaching a correct diagnosis without the aid of genetic tests (Morimoto et al., 2019; Wirschell et al., 2013).

It is known that episodes of neonatal respiratory distress at full‐term birth are common in PCD (Knowles et al., 2016; Mullowney et al., 2014), but are less frequent in Japanese PCD patients as a whole. In addition, congenital heart disease and otitis media are also not commonly observed in Japan (Amitani et al., 1990; Inaba et al., 2019). This may indicate two possibilities: first, pediatricians are mostly unaware of PCD until adulthood; or second, alternatively, signs and symptoms of PCD in Japanese are relatively mild and somewhat controllable by early treatment with antibiotics in infancy or childhood. Mild clinical signs and symptoms depending on a particular genotype have recently been reported in PCD caused by *RSPH1* mutations (Knowles et al., 2014).

In the present study, using a multiplexed PCR‐based method, we found a large homozygous deletion spanning exons 1–4 of *DRC1* in two of 105 patients with definite DPB according to the current clinical criteria, and another patient with recurrent airway infections not fulfilling the criteria. In the subsequent analysis of the healthy Japanese population, 0.2% of alleles had a large deletion with identical breakpoints, expecting 0.0004% as homozygotes by the Hardy‐Weinberg principle. If prevalence of Japanese PCD is estimated at 1:20,000 ( = 0.005%) (Takeuchi et al., 2018), PCD caused by this deletion in *DRC1* accounts for 8% ( = 0.0004/0.005), indicating that this *DRC1* mutation may be the most common cause of PCD in Japan, since, so far, no mutation hotspots of known PCD genes have been identified in Japan (Takeuchi et al., 2018). In the two patients heterozygous for the same deletion, no other pathogenic or likely pathogenic variants were found in any exons and exon‐intron boundaries of *DRC1*. Further investigation would be necessary to determine whether pathogenic variants for compound heterozygosity are hidden in unscreened regions of the gene.

So far, ciliary defects in PCD can be classified into five main ultrastructural and other phenotypes as follows: the absence of outer dynein arms (ODAs), due to mutations in genes encoding ODA subunits; the absence of both ODAs and inner dynein arms (IDAs), caused by mutations related to dynein arm assembly; the absence of IDAs with microtubule disorganization, related to *CCDC39* and *CCDC40*, essential to the docking of IDAs; an intermittent absence of the central pair and radial spoke defects in mutations of the radial spoke proteins; and an apparent absence of nexin link or even no detectable abnormalities on electron microscopy (EM) analysis but related to mutations of a subunit of nexin‐dynein regulatory complexes (N‐DRCs), involved in the connection between the IDA, radial spokes, and outer doublets (Shapiro & Leigh, 2017). PCD causative mutations in this fifth category have recently been reported in three human genes, *DRC1*, *CCDC65* (*DRC2*), and *GAS8* (*DRC4*) of the N‐DRC subunits (Austin‐Tse et al., 2013; Horani et al., 2013; Jeanson et al., 2016; Morimoto et al., 2019; Olbrich et al., 2015; Wirschell et al., 2013).

Although homozygous nonsense mutations in the human *DRC1* gene were clearly demonstrated in 2013 with immunofluorescence staining results of N‐DRC subunits in four patients as one type of PCD (Wirschell et al., 2013), the subsequent case report has not yet been published. Strikingly, all of the 13 patients with N‐DRC subunit defects (*DRC1*, *CCDC65*, and *GAS8*) reported previously had situs solitus, presumably because subtle beating pattern abnormalities caused by these defects would be efficient enough to propel the extra‐embryonic fluid toward the left side of the embryo (Jeanson et al., 2016; Shapiro & Leigh, 2017). All of our cases without situs inversus may support the above possibility, though more case studies have been awaited (Morimoto et al., 2019). Furthermore, as compared with other forms of the disease, including those with *CCDC39* and *CCDC40* mutations (Davis et al., 2015), *DRC1* mutations may have a mild clinical phenotype, at least in Japanese, with a delayed onset of symptoms and long survival presented in our report, considering a seemingly adult onset of the disease. It is not known yet whether the frequency of infertility caused by such subtle beating pattern defect is lower than that of other structural defects with ciliary immotility.

This type of PCD is not readily identified by either high‐speed video‐microscopy or EM analysis (Olbrich et al., 2015; Shapiro & Leigh, 2017) but rather associated with a secondary IDA defect due to inflammatory changes or artifact (O'Callaghan, Rutman, Williams, & Hirst, 2011). Nasal nitric oxide (NO) levels have been reported to be low for all N‐DRC defects, including *DRC1* mutations (Morimoto et al., 2019; Wirschell et al., 2013). This finding is worthwhile because nasal NO is a useful noninvasive test in suspecting PCD (Leigh et al., 2013), even though the patients do not reveal any specific findings clinically. DPB also shows relatively low nasal NO levels in one report (Nakano et al., 2000), but a future comparison study may determine whether an appropriate cutoff value distinguishes most PCD types from classical DPB, after excluding extremely rare CF in Asia (Yamashiro et al., 1997).

Based on the references (Amitani et al., 1990; Austin‐Tse et al., 2013; Homma et al., 1983; Horani et al., 2013; Jeanson et al., 2016; Keicho & Kudoh, 2002; Olbrich et al., 2015; Wirschell et al., 2013), we summarized clinical similarities and possible differences between classical DPB and PCD with N‐DRC mutations in Table S5. Clinical signs and symptoms are similar to each other, but classical DPB has been regarded as an adult disease; lower respiratory symptoms are commonly observed in people in their 40s or 50s, even though chronic rhinosinusitis is experienced often from childhood, and abnormalities at birth have not been reported yet.

Pulmonary lesions on CT images are not easily distinguished between the two diseases when bilateral centrilobular nodules are observed. It is not known whether bronchiectasis predominantly affecting the middle lobe or lingula is more common in PCD than DPB (Kennedy et al., 2007). Pathologically, Homma previously reported that main pulmonary lesions shown as diffuse centrilobular nodules on chest CT (Amitani et al., 1990) are membranous bronchiolitis in PCD with Kartagener triad, distinct from respiratory bronchiolitis in classical DPB (Homma et al., 1999); however, no pathological reports on PCD caused by mutations of an N‐DRC subunit have been published yet. In classical DPB, cilia function may be secondarily affected by chronic airway inflammation (Amitani et al., 1990), but the motion of sperm is not affected, and consequently, infertility is usually not observed.

In the treatment of DPB, long‐term macrolide therapy has strikingly improved the prognosis (Kudoh, Azuma, Yamamoto, Izumi, & Ando, 1998), whereas, in PCD, the effect seems to be variable (Knowles et al., 2016). In the present study, treatment effects were hardly observed in one of the two patients. Considering similarities and differences of the two diseases and impaired mucociliary clearance, pathogenesis including disease susceptibility genes of DPB should also be revisited. This is also important because a major disease susceptibility gene of DPB has been hypothesized in a mucin gene cluster between the HLA‐A and HLA‐B loci (Hijikata et al., 2011; Keicho & Hijikata, 2011; Keicho et al., 2000), but has not been specified yet.

Since this was a retrospective analysis, we used a panel of DNA samples and their corresponding data records, and could not access patients' detailed information or other test results, which limited our study. No parents' or sibling's DNA was, thus, obtained. Although a major JPT haplotype that carries the deletion in *DRC1* was a minor haplotype in the CEU population, a disease‐specific haplotype exclusively in Asians was not determined. To characterize patients with PCD in Asia further, prospective studies on sinopulmonary diseases including nasal NO, genetic tests, and ideally, immunofluorescence staining of cilia subunits, should be conducted.

We identified the same homozygous deletions in *DRC1* in Japanese patients initially diagnosed as having DPB and demonstrated that this mutation may be one of the common causes of PCD in Japan. Since this type of PCD is not readily identified by high‐speed video‐microscopy or EM analysis, nasal NO measurement with appropriate genetic testing would be useful in differential diagnosis. Investigation on sinopulmonary diseases including search for founder genetic mutations that cause ethnic variants of PCD may be worth considering in Asian populations.

## CONFLICT OF INTEREST

There are no conflicts of interest to declare.

## Data Availability

Data available on request from the authors.
