# Supplementary figures and images for: Primary ciliary dyskinesia caused by a large homozygous deletion including exons 1–4 of DRC1 in Japanese patients with recurrent sinopulmonary infection
Source: Mol Genet Genomic Med. 2019 Nov 8;8(1):e1033. doi: 10.1002/mgg3.1033 (PMC6978274; doi:10.1002/mgg3.1033)

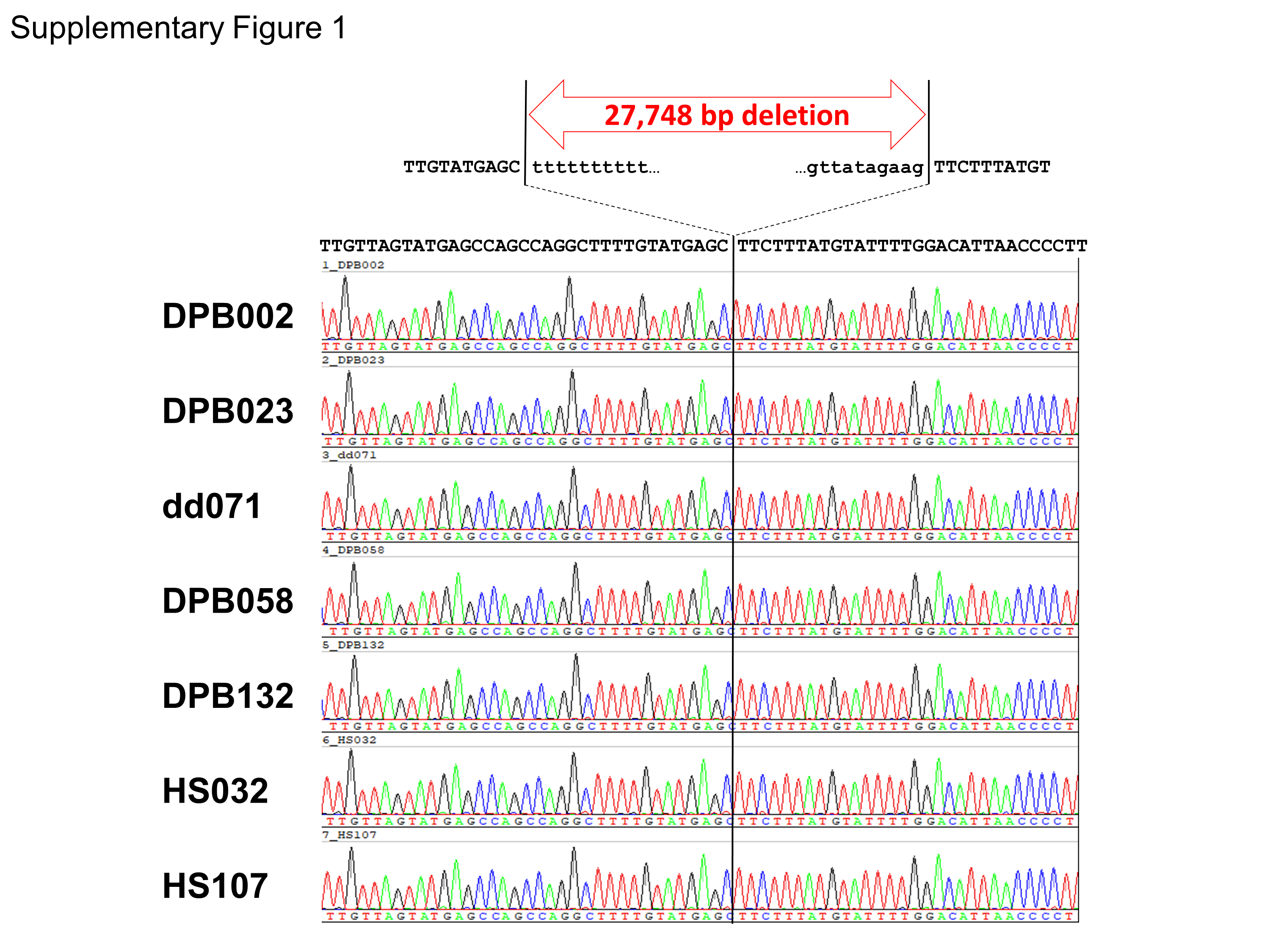

Supplement: Supplementary file 1 [file MGG3-8-e1033-s001.TIF]
